# Main facial anatomical concerns of dentists to perform botox injections

**DOI:** 10.4317/jced.62917

**Published:** 2025-08-01

**Authors:** Paulo Henrique Ferreira Caria, Jéssica da Silva Sousa, Wagner José Fávaro

**Affiliations:** 1 Associated Professor - Department of Structural and Functional Biology, Institute of Biology, State University of Campinas - UNICAMP; 2 Undergraduate Student – Piracicaba Dental School - State University of Campinas – UNICAMP

## Abstract

**Background:**

The increasing demand for minimally invasive aesthetic procedures, particularly botulinum toxin type A (BTX-A) injections, has expanded the scope of dental practice to include facial aesthetics. However, the safe and effective administration of BTX-A requires a thorough understanding of facial anatomy, including the topographic relationships of muscles, skin layers, and vascular structures. This cross-sectional study aimed to identify the primary anatomical concerns and knowledge gaps among dentists performing BTX-A injections.

**Material and Methods:**

A structured questionnaire was distributed to 316 dentists, assessing their confidence and knowledge regarding facial anatomy and BTX-A application.

**Results:**

A total of 62% of participants had specialized training, while 37% had attended BTX-A-specific courses. Notably, professionals with specialized training demonstrated significantly greater confidence and anatomical knowledge compared to their non-specialized counterparts. Key areas of concern included the corrugator supercilii, levator labii superioris alaeque nasi, orbicularis oculi, frontalis, and platysma muscles, as well as the facial artery’s location and depth. Over 80% of respondents reported limited or insufficient knowledge in these areas, highlighting a critical need for enhanced anatomical education and practical training.

**Conclusions:**

The study underscores the importance of integrating facial anatomy and injection techniques into dental curricula and continuing education programs to ensure patient safety and optimize treatment outcomes. By addressing these knowledge gaps, dentists can confidently expand their practice to include BTX-A applications, contributing to the growing field of orofacial harmonization.

** Key words:**Botulinum toxin type A, Facial anatomy, Dental practice, Cosmetic Dentistry; Esthetic Dentistry.

## Introduction

Over recent decades, the global demand for aesthetic treatments has increased significantly across diverse age groups, genders, and ethnicities [1]. Non-surgical and minimally invasive facial procedures are widely sought for their ability to reduce the visible signs of aging, prevent their progression, and improve individuals’ quality of life [2]. Among these procedures, Botulinum toxin type A (BTX-A), commercially known as Botox, remains the most frequently requested and performed non-surgical cosmetic intervention worldwide [3,4].

The widespread use of BTX-A is primarily attributed to its efficacy in diminishing facial wrinkles and fine expression lines, thereby contributing to a more youthful appearance. Its reversible nature, combined with a broad spectrum of cosmetic and therapeutic indications, has made BTX-A a highly preferred agent in aesthetic medicine [5-7].

In recent years, the professional scope of dentistry has expanded beyond traditional oral health care to encompass facial aesthetic procedures. Within this evolving landscape, the use of BTX-A by dental professionals has gained prominence. However, safe and effective application of BTX-A requires not only technical competence but also a thorough understanding of facial anatomy. Dentists must therefore undergo comprehensive training, demonstrate clinical proficiency, and adhere to principles of patient safety and ethical practice.

The musculature responsible for facial expression possesses distinctive anatomical characteristics that differentiate it from other skeletal muscle groups—most notably, its integration with the superficial musculoaponeurotic system (SMAS) and direct insertions into the dermis [5,8]. These muscles are often located in close proximity, within the same anatomical plane, with some positioned more deeply. Such anatomical arrangements facilitate potential diffusion of BTX-A to unintended targets, increasing the risk of undesirable outcomes.

The growing popularity of non-surgical aesthetic procedures has led to a proliferation of training and certification programs for dentists, including numerous online offerings promoted through social media platforms. Despite this, many programs provide limited practical experience and insufficient instruction in applied facial anatomy, contributing to practitioner uncertainty and a higher incidence of technical errors and complications [9]. As a result, adverse effects following BTX-A injections vary according to the treated area and may include facial asymmetry, eyebrow ptosis, blepharoptosis, brow ptosis, impaired eyelid function, ocular sensory disturbances, lip asymmetries, and lower facial muscle imbalances [10,11].

Given these concerns, ensuring practitioner competence and anatomical literacy is critical for the safe administration of BTX-A in aesthetic dentistry. The present study aims to identify the principal anatomical challenges and uncertainties reported by dentists in the context of BTX-A injections in the facial region.

## Material and Methods

This cross-sectional study was approved by the University Ethics Committee, protocol number CAAE: 74932917.9.0000.5418. Participation in this study was voluntary, and informed consent was obtained from all volunteers.

- Sample 

The volunteers in this research were generalist and/or specialist dentists from different areas, who have been working in dental clinics and/or private practices for at least 1 year. We used the sampling formula for qualitative variables (n = (z1-α/2)2 • p(1-p) / d2), where Z1-α/2 = standard normal variate (at 5% type 1 error (*P* < 0.05) it is 1.96) and *p* = expected proportion in the population based on the number of active dentists in the state of São Paulo. Where d = absolute error or precision and was decided by the researcher [12]. In this study, it was assumed that α (type I error) was 0.05 and precision was 5%; thus, the sample size was calculated to be 320. However, considering probable sample loss, recruitment of 420 questionnaires was planned.

- Study Design 

For the conduct of this study, a physical questionnaire was developed based on Cluster 6 of ALE (Anatomy Learning Experience Questionnaire) [13] and presented to all professionals who enrolled in various postgraduate courses at the State University of Campinas. The questionnaire was divided into two parts; the first part contained questions related to professional profile, and the second part focused on the professional’s confidence and safety in performing botulinum toxin injections on their patients [14,15].

The questions were formulated in a simple and objective manner to identify the main concerns about facial anatomy and its possible relationships with the dentists’ years of experience and training courses. The questionnaire was reviewed beforehand by professors and researchers in the field of Anatomy and Facial Harmonization to ensure the validity and coherence of the question content. Subsequently, it was distributed to the dentists/volunteers with a request for same-day completion to avoid consultations or biases in the responses.

The first section of the questionnaire collected demographic and professional background information, including gender, age, years of university education, specialization status (formal postgraduate programs in any dental specialty) and participation in any refresher courses or training related to BTX-A use (short-term continuing education programs focused exclusively on the clinical application of botulinum toxin type A), along with the time duration of such training.

The second part consisted of eighteen questions regarding the professional’s confidence and safety related to their knowledge of facial anatomy, specifically focusing on BTX-A application on the face, with response options being: Very, somewhat, or not at all.

Dentists who had already completed a specialization course in Orofacial Harmonization were excluded from the study. Descriptive statistics were applied to the completed questionnaires to analyze response proportions. Variability analysis was conducted to assess data dispersion, and the Spearman test was used to evaluate the monotonic association between variables.

## Results

Out of 420 distributed self-response questionnaires, we obtained complete responses from 316 participants, yielding a response rate of 75.2%. A significant association was found between the independent variable and the volunteers’ responses (*p* < 0.05).

Descriptive statistical analysis was conducted to examine respondents’ profiles and their self-perceived knowledge of facial anatomy for botulinum toxin application. The questionnaire consisted of two sections. The first section aimed to characterize the participants’ professional profiles, including gender, age, years of education, specialization courses, and prior training on botulinum toxin use for dental purposes.

 Table 1. Representative Table of the percentage relative to the profile of volunteers who sought an update/training course in botulinum toxin for dentists.

Women comprised 76% of the study participants, while men accounted for 24%. The mean age of participants was 36 ± 3.2 years, ranging from 26 to 49 years. The average time since graduation was 13 ± 6.8 years, with experience levels varying from recent graduates with 2 years of practice to seasoned professionals with up to 27 years.

A majority (62%) had completed some form of specialization before pursuing training in botulinum toxin, while 38% were general practitioners without specialization. Additionally, 37% had attended a course specifically on botulinum toxin use for dentists, with an average course duration of 16 hours. Conversely, 73% had not received any formal training on the subject.

Analysis of the relationship between the demographic data (Part 1) and participants’ responses (Part 2) revealed that female participants (58.8%) and those with more extensive training (61.7%) were more likely to respond “a lot” or “a little” compared to male participants (41.2%) and those with less training (38.3%). Regarding age, participants who answered “a lot” or “a little” had a mean age of 28 ± 2.2 years.

Furthermore, respondents with specialization had a higher percentage of “a lot” and “a little” responses (69.2%) across all 18 questions in Part 2, compared to those without specialization (30.8%). Similarly, participants who had attended a botulinum toxin course for dentists had a greater proportion of “a lot” and “a little” responses (78.7%) compared to those who had never taken such a course (21.3%).

PART 2. Considering that botulinum toxin is administered via intramuscular injection, please answer the following questions based solely on your anatomical knowledge of this procedure.

Questions

1. Do you know the muscles of facial expression?

2. Do you know the chewing muscles?

3. Do you feel confident injecting botulinum toxin into your patients’ faces??

4. Do you feel confident injecting botulinum toxin into the frontalis muscle??

5. Do you feel confident injecting botulinum toxin into the procerus muscle?

6. Do you feel confident injecting botulinum toxin into the orbicularis oculi muscle?

7. Do you feel confident injecting botulinum toxin into the corrugator supercilii muscle?

8. Do you feel confident injecting botulinum toxin into the buccinator muscle?

9. Do you feel confident injecting botulinum toxin into the depressor labii inferioris muscle?

10. Do you feel confident injecting botulinum toxin into the depressor anguli oris muscle?

11. Do you feel confident injecting botulinum toxin into the orbicularis oris muscle?

12. Do you feel confident injecting botulinum toxin into the levator labii superioris alaeque nasi muscle?

13. Do you feel confident injecting botulinum toxin into the masseter muscle?

14. Do you feel confident injecting botulinum toxin into the temporalis muscle?

15. Do you feel confident injecting botulinum toxin into the platysma muscle?

16. Do you feel confident injecting botulinum toxin into the major salivary glands?

17. Do you feel confident in avoiding injection of botulinum toxin into blood vessels?

18. Do you know the skin layers and planes you pass through before reaching the muscle segment to apply botulinum toxin??

19. Do you know the location of the facial nerves to prevent complications during botulinum toxin injection?, (Figs. 1,2).

**Figure 1:**
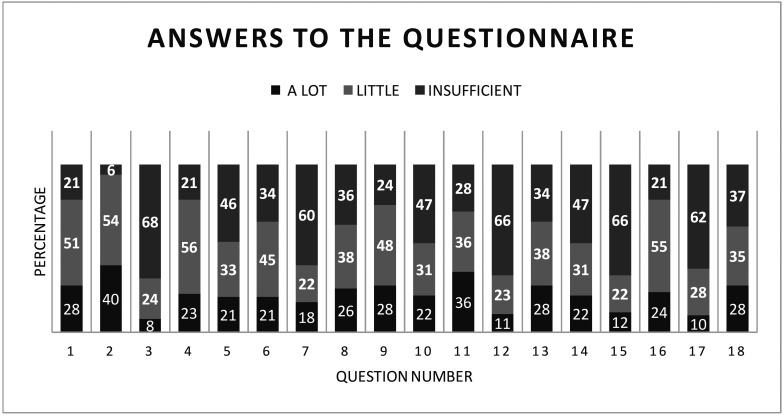
Graphic Representation of the percentage relative to answer of questions about BTA injection. (Possible answers: a lot, little, insufficient).

**Figure 2:**
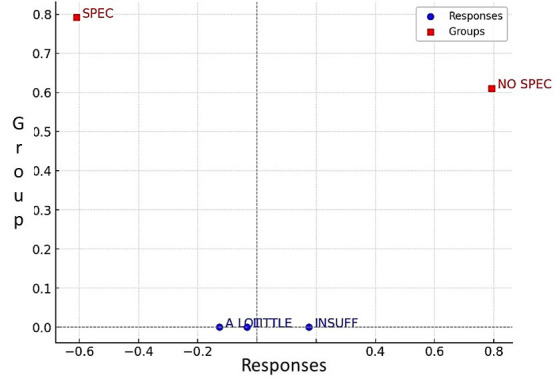
Variability, Group Associations, and Study Responses.

The total inertia value of 0.15 suggests a moderate association structure. The SPEC group exhibits a strong association with A LOT responses, whereas the NO SPEC group is strongly linked to INSUFF responses. LITTLE responses show a moderate association with NO SPEC but also appear relatively close to SPEC along the second dimension.

Spearman’s correlation test between the study variables. The Table presents the correlation coefficients between different response categories and groups analyzed, ( Table 2).

The Spearman test was used to evaluate the monotonic association between variables, measuring both the strength and direction of their relationship. Positive correlation values indicate a direct relationship, where an increase in one variable corresponds to an increase in the other. Conversely, negative correlation values indicate an inverse relationship, where an increase in one variable corresponds to a decrease in the other.

Statistical significance was determined based on *p-value*s, which guided the interpretation of the results. The strength of correlations was classified as follows: Weak: rs < 0.3; Moderate: 0.3 ≤ rs < 0.7; Strong: rs ≥ 0.7.

## Discussion

Botulinum toxin type A (BTX-A) is widely employed for both aesthetic and therapeutic purposes and remains the most frequently performed non-surgical facial procedure globally [16]. Its popularity stems from its minimally invasive nature, short recovery time, and minimal discomfort or scarring [17]. Consequently, an increasing number of dentists have incorporated BTX-A into their clinical practice alongside conventional dental procedures.

A robust understanding of facial anatomy is becoming an essential competency for modern dental practitioners. Dentists of the future are expected to extend their role beyond traditional oral healthcare providers to encompass aspects of facial aesthetics and patient-centered care, integrating clinical and even psychological perspectives. The Council of European Dentists (CED) acknowledges this shift, emphasizing the need for enhanced aesthetic and anatomical expertise in orofacial soft tissue management [18]. In this context, our study contributes significantly, for both professors and dentists, by identifying the key anatomical challenges and uncertainties dentists face when administering BTX-A, thus underscoring the need for continuing education and advanced anatomical training.

Despite BTX-A being used in dentistry for over five years, no prior studies have systematically explored the specific uncertainties professionals face in its application. Our findings highlight a critical gap in anatomical knowledge, particularly among non-specialist practitioners. Overall, professionals with specialization exhibited significantly better anatomical understanding (78.7% vs. 21.3%). Younger dentists (mean age: 28 years) also reported higher confidence levels, potentially due to more recent academic exposure or improved teaching methodologies [19].

Statistical analyses, including Simple Correspondence Analysis and the Spearman test, corroborated that specialists possessed superior anatomical knowledge, while non-specialists reported lower confidence underscoring the value of including facial anatomy education in postgraduate training [20].

Although BTX-A is generally safe and effective, the growing number of practitioners using it has coincided with a proportional rise in complications [21]. Adverse events typically result from three factors: patient-related variables, product-related issues, and technique-related errors particularly insufficient anatomical knowledge and lack of experience [22]. While patient and product considerations (e.g., hypersensitivity, medical history, neuromuscular conditions, or improper dosage) are critical [23,24], our study primarily addresses technique-based shortcomings.

Enhanced anatomical education plays a pivotal role in reducing complication rates and improving patient outcomes in dental BTX-A applications. As demonstrated in our results, a thorough understanding of the spatial arrangement, depth, and functions of facial muscles, nerves, and vasculature enables clinicians to perform injections with greater accuracy and safety. Conversely, insufficient anatomical knowledge is linked to complications such as facial asymmetry, unintended muscle paralysis, eyelid ptosis, and vascular injury [11,21,24]. Incorporating detailed anatomical instruction into dental education and specialization programs improves practitioners’ ability to identify anatomical variations, apply precise techniques, and personalize treatments [9,25], thereby minimizing complications and enhancing both aesthetic and functional outcomes.

More than 80% of responses to Questions 3, 7, 12, 15, 17, and 18 fell under the “little” or “insufficient” knowledge categories, reflecting considerable professional insecurity surrounding BTX-A use. The face is composed of seven primary anatomical layers: 1) skin, 2) superficial fat, 3) superficial muscular aponeurotic system (SMAS), (4) muscle, 5) vasculature, 6) deep fat, and 7) bone [25]. A comprehensive understanding of these layers is essential for minimally invasive procedures such as BTX-A injections and dermal fillers. Previously undervalued in dental education, this knowledge is now critical for patient safety and procedural success.

Mastering the three-dimensional organization of facial structures facilitates precise needle positioning and injection into specific muscles. Our study found that most participants demonstrated limited or insufficient knowledge in this area. Despite its importance, this topic is often absent from undergraduate medical and dental curricula. Each facial layer has unique morphological and clinical characteristics and is differentially affected by aging. Understanding these layers allows clinicians to perform personalized, safe, and effective aesthetic interventions.

A notable knowledge gap was highlighted regarding the corrugator supercilii muscles (Question 7), which are thin, narrow bands located beneath the frontalis and orbicularis oculi muscles. These muscles originate from the medial supraorbital ridge and insert into the medial brow, producing vertical glabellar lines when contracted. They have two heads: a transverse head, which pulls the eyebrows medially, and an oblique head, which lowers them. Their contraction produces vertical wrinkles on the forehead. Together with the procerus muscle, they play a key role in expressions associated with anger, worry, or confusion [26,27]. Due to their anatomical depth and variability, they are often misunderstood, increasing the risk of injection errors. Incorrect placement of BTX-A in the frontalis instead of the corrugator can lead to the “Mephisto effect,” characterized by an arched or sinister-looking eyebrow [28].

Similarly, limited understanding was observed regarding the levator labii superioris alaeque nasi (LLSAN) muscle (Question 12). This muscle, crucial in the pathophysiology of the gummy smile (GS), originates from the frontal process of the maxilla and divides into two fascicles inserting into the upper lip and nasal ala. Accurate injection into the LLSAN is essential for GS correction, a common aesthetic concern associated with excessive gingival display (>2 mm) [30-32]. BTX-A offers a safe, minimally invasive alternative to surgical interventions in these cases. However, due to the anatomical complexity of the LLSAN, practitioners frequently report difficulty performing injections with confidence [20,29]. Over 80% of participants indicated insufficient knowledge about the frontalis (Question 4) and orbicularis oculi (Question 6) muscles—both integral to facial aesthetics. Such responses suggest a lack of confidence even among professionals with BTX-A experience. Without precise anatomical knowledge, adverse outcomes such as eyebrow asymmetry, ptosis, or ectropion may result [21,33].

The platysma muscle (Question 15) also emerged as a source of significant practitioner uncertainty. This thin, broad muscle spans the neck and lower face and contributes to expressions and jawline definition. BTX-A injections targeting platysmal bands are increasingly popular for facial contouring but require detailed knowledge of subdermal anatomy to avoid complications such as dysphonia, dysphagia, and facial asymmetry [34,35]. Given that our specialist group included dentists from non-aesthetic fields, their knowledge gaps mirrored those of non-specialists, reflecting the novelty of orofacial harmonization as a formal specialty.

A thorough understanding of facial vascular anatomy is equally crucial. Although vascular complications from BTX-A are rare, they are increasingly reported in the literature [36]. A review of adverse events from 1965 to 2018 identified 1,459 cases, most associated with BTX-A, with only a small percentage linked to soft tissue fillers [37]. Vascular events may present as early cutaneous changes or progress to severe outcomes such as necrosis, blindness, stroke, or pulmonary embolism [38]. The facial venous network’s complexity and variability further elevate these risks, especially in anatomically sensitive zones such as the glabella, nasal dorsum, and periorbital region. The lack of venous valves in key facial veins can enable retrograde flow of injectables, complicating diagnosis and treatment [39,40].

The facial artery, a branch of the external carotid artery, follows a tortuous course with variable depth and critical branches. High-risk regions where the artery becomes superficial—such as the mandibular border, nasolabial fold, and lips—are particularly prone to complications during aesthetic procedures. Mastery of its topography, combined with safe techniques such as aspiration prior to injection, is essential for avoiding vascular compromise [41-43].

## Conclusions

This study identified key anatomical challenges faced by dental professionals in the application of botulinum toxin type A (BTX-A), particularly regarding the layered structure of the face and the topography of critical muscles and blood vessels. Practitioners with specialized training exhibited significantly better anatomical knowledge, which correlated with increased confidence and competence in aesthetic procedures. While the integration of BTX-A into dental practice opens new avenues for patient care and professional growth, achieving optimal outcomes demands a strong foundation in facial anatomy. Expanding anatomical education within dental training both at the undergraduate and postgraduate levels is essential to enhance patient safety, reduce complications, and promote best practices in facial aesthetics.

## Figures and Tables

**Table 1:** Respondents profile.

Age (Mean±SD)	36 ± 3.2
Sex	n (%)
Female	141 (76)
Male	45 (24)
How long have you been in practice?	13 ± 6.8 anos
Do you have any specialization?	
Yes	116 (62)
No	70 (38)
Have you attended any courses on the use of botulinum toxin for dentists?	
Yes	69 (27)
What is the duration?	MD:16h
No	31 (73)

**Table 2:** Spearman’s correlation test between the study variables. The table presents the correlation coefficients between different response categories and groups analyzed.

Variable 1	Variable 2	Coeficiente de Spearman	p- Value
A LOT	SPEC	0.628	0.004
A LOT	NO SPEC	0.570	0.011
NO SPEC	SPEC	0.688	0.001
LITTLE	A LOT	0.525	0.021
SPEC (2)	NO SPEC	-0.535	0.018
INSUFFICIENT	A LOT	-0.803	<0.001
INSUFFICIENT	LITTLE	-0.911	<0.001
SPEC (2)	SPEC	-0.612	0.005
SPEC (2)	NO SPEC	-0.456	0.050

## Data Availability

The datasets used and/or analyzed during the current study are available from the corresponding author.
